# Adrenal Dysfunction in Portal Hypertensive Rats with Acute Hemorrhage

**DOI:** 10.1371/journal.pone.0092093

**Published:** 2014-03-14

**Authors:** Fa-Yauh Lee, Sun-Sang Wang, Ming-Hung Tsai, Hui-Chun Huang, Han-Chieh Lin, Shou-Dong Lee

**Affiliations:** 1 Faculty of Medicine, National Yang-Ming University, Taipei, Taiwan; 2 Division of Gastroenterology, Department of Medicine, Taipei Veterans General Hospital, Taipei, Taiwan; 3 Department of Medical Affair and Planning, Taipei Veterans General Hospital, Taipei, Taiwan; 4 Division of Gastroenterology, Department of Medicine, Cheng Hsin General Hospital, Taipei, Taiwan; 5 Division of Digestive Therapeutic Endoscopy, Chang Gung Memorial Hospital, Taipei, Taiwan, Chang Gung University College of Medicine, Taoyuan, Taiwan; Kaohsiung Medical University Hospital, Kaohsiung Medical University, Taiwan

## Abstract

Nitric oxide (NO) participates in shock and poorer portal hypotensive effect to vasoconstrictors in portal hypertension with hemorrhage, the so-called splanchnic hyposensitivity. Relative adrenal insufficiency accompanies hemorrhagic shock and is found in liver disease, the ‘hepatoadrenal syndrome’, but the relevant interactions remain unsettled. Portal hypertensive rats were induced by partial portal vein ligation (PVL). Experiments were performed on the 14^th^ day post PVL: (I) ACTH stimulation test for rats without or with hemorrhage; (II) Glypressin response (mean arterial pressure, MAP; portal pressure, PP) in rats (a) without hemorrhage or with hemorrhage, injected with (b) distilled water (DW), (c) dexamethasone 3 mg/kg; (III) To survey the dose-dependent effects of glucocorticoid without being confounded by endogenous adrenal hormone, glypressin response was surveyed in PVL rats with adrenalectomy: (a) without hemorrhage or with hemorrhage, injected with (b) DW; (c) dexamethasone 3 mg/kg; (d) dexamethasone 5 mg/kg. Plasma tumor necrosis factor-α (TNF-α) concentrations and abdominal aorta (AA), superior mesenteric artery (SMA) NO synthases (NOS) mRNA expressions were determined. The results showed that ACTH induced corticosterone release similarly in PVL rats with or without hemorrhage. In bleeding PVL rats, dexamethasone (1) down-regulated AA NOS and enhanced glypressin-induced MAP elevation; (2) did not influence glypressin-induced PP reduction; (3) reduced TNF-α. In bleeding PVL and adrenalectomized rats, high-dose dexamethasone (1) down-regulated AA/SMA NOS; (2) enhanced glypressin-induced MAP elevation and PP reduction; (3) reduced TNF-α. In conclusion, bleeding portal hypertensive rats failed to enhance corticosterone release, suggesting a relative adrenal insufficiency. High-dose dexamethasone reversed systemic hypotension and splanchnic hyporesponsiveness to glypressin in adrenalectomized PVL rats accompanied by TNF-α and NOS down-regulation, suggesting the importance of adequate adrenocorticoid supplement in portal hypertension with hemorrhage and adrenal dysfunction.

## Introduction

Liver cirrhosis and portal hypertension complicated by ruptured gastroesophageal varices may lead to hemorrhagic shock, resulting in events as (i) reduction of blood pressure; (ii) endogenous vasoconstrictors release in an attempt to maintain blood pressure; (iii) vascular hyporeactivity to vasoconstrictors. Vasopressin, a vasoconstrictor that decreases portal pressure (PP) via constricting mesenteric vasculatures [1], has been used to control variceal bleeding. However, recent studies have shown that vasopressin administrated during hemorrhage is less effective than that used in those without hemorrhage in cirrhosis and portal hypertension (the so-called vascular hyposensitivity) [2]. Similar conditions were also found in glypressin, a long-acting vasopressin analogue [3]. The exact cause is not fully understood but may be related to the excessive synthesis of endogenous vasopressors [4] and/or the secretion of mediators affecting vasoconstriction during hypovolemia [5].

Nitric oxide (NO), a vascular endothelium-derived vasodilatory substance, plays a key role in the pathogenesis of vascular hyporesponsiveness in hemorrhagic shock [6]. It has been found that NO synthesis blockade in partial portal vein ligation (PVL)-induced portal hypertensive rats with hemorrhage alleviated the glypressin hyposensitivity [3]. NO synthase (NOS) exists in either constitutive or inducible isoform. While the constitutive form (eNOS) may be activated by mechanical factors [7], the inducible form (iNOS) can be triggered by endotoxin and cytokines such as tumor necrosis factor-α(TNF-α) [8].

Critical illness is accompanied by the hypothalamic-pituitary-adrenal (HPA) axis activation highlighted by increased serum cortisol levels [9]. This is a crucial component of the host's adaptation to severe stress, since corticosteroids restore vascular tone by potentiating vascular responses to endogenous and exogenous vasoconstrictors [10]. It has been demonstrated that hydrocortisone corrected the vascular hyporeactivity in patients with septic shock [11]. The beneficial effects may be resulted from the inhibition of cytokines and NO [12], followed by the reverse of vasocontractile capability and maintenance of blood pressure.

Recently, the concept of critical illness–related corticosteroid insufficiency (CIRCI) has been introduced to describe a subnormal adrenal response to adrenocorticotropin in severe illness, in which the cortisol levels, even though high in terms of absolute value, are inadequate to alleviate the stress-related shock [9]. It is worth noting that patients with adrenal insufficiency share similar hemodynamic features with cirrhotic patients, namely increased cardiac output, decreased peripheral vascular resistance, decreased mean arterial pressure (MAP), and hypo-responsiveness to vasopressors [13], [14]. Furthermore, the hyperdynamic circulation is mainly mediated by NO [15]. Our group and others have shown that adrenal insufficiency is common in critically ill patients with liver diseases and carries prognostic significance [16], [17]. These abnormalities have been described in the absence of clinical sepsis, raising the possibility that adrenal insufficiency arising in portal hypertension may be a different entity to that observed in septic shock. The distinct findings led to the term as ‘hepatoadrenal syndrome’ [18].

Hemorrhagic shock and ischemia–reperfusion consequences are leading causes of non-septic inflammatory reaction. As with septic patients [19], an attenuated cortisol response to corticotropin stimulation has been reported following hemorrhagic shock and was associated with an increased need for vasopressor therapy [20]. This vasopressor dependency may be attributed to the NO-mediated vascular hyporeactivity following hemorrhage.^6^ Furthermore, dexamethasone corrected vascular hyporeactivity by inhibiting NO synthesis in the setting of hemorrhagic shock [6], [21]. Despite the accumulating data in this area, the role of adrenocorticoid in vascular hyporeactivity to vasopressor in portal hypertension with hemorrhage is unrecognized and underestimated.

Taken together, we hypothesized that the HPA axis activation during acute hemorrhage might be compromized in portal hypertension, resulting in hemodynamic impairment that could be corrected by glucocorticoid. The study aimed to survey: (i) the adrenal function; (ii) the systemic and splanchnic vascular reactivity to glypressin in acute hemorrhage; (iii) the circulating TNF-α levels and systemic (abdominal aorta, AA) and splanchnic (superior mesenteric artery, SMA) vascular NOS expressions; (iv) the effects of dexamethasone on the aforementioned parameters. To exclude the potential interference exerted by endogenous adrenal hormone and to test the exact action of exogenous adrenocorticoid hormone, another series of the same experiments performed on PVL rats subjected to adrenalectomy were performed.

## Methods

### Animal model: partial portal vein ligation (PVL) without or with adrenalectomy

Male Sprague-Dawley rats (280–300 g) were used. The rats were allowed free access to food and water. All rats were fasted for 12 hours before the operation. Prehepatic portal hypertension was created by partial portal vein ligation (PVL) as previously described [22]. Experiments were performed 14 days after PVL.

To further demonstrate the dose-dependent effect of glucocorticoid in portal hypertension with hemorrhage and to avoid the potential interference exerted by endogenous adrenal hormone, bilateral adrenalectomy was performed on the same day of PVL as previously described [23]. Adrenalectomized animals were given saline instead of tap water thereafter to compensate for the absence of mineralocorticoid regulation of sodium. The adequacy of the adrenalectomy was ascertained at the end of the experiments by careful autopsy examination.

This study was approved by Chang Gung Memorial Hospital Institutional Animal Care and Use Committee (IUACU, Approval No. 2009121508). All animals received humane care according to the criteria outlined in the “Guide for the Care and Use of Laboratory Animals” prepared by the National Academy of Sciences and published by the National Institutes of Health (NIH publication 86–23 revised 1985).

### Measurement of systemic and portal hemodynamics

Mean arterial pressure and PP were measured by catheterization of right femoral artery and ileocolic vein, respectively with a Spectramed DTX transducer (Spectramed Inc., Oxnard, CA). Continuous recordings of mean arterial pressure (MAP), heart rate (HR) and portal pressure (PP) were performed on a multichannel recorder (model RS 3400, Gould Inc., Cupertino, CA) [24]. The external zero reference was placed at the level of the mid-portion of the rat.

### ACTH-stimulated release of corticosterone

At 1 hour after the completion of hemorrhage and transfusion, 100 μg of porcine corticotropin (ACTH (1–39)) in 0.2 ml isotonic sodium chloride was administered intravenously. Blood samples were taken before and 30 minutes after corticotropin challenge as previously described [25]. The corticosterone response is defined as the difference between the baseline and peak corticosterone levels.

### Plasma corticosterone and TNF-α concentration determinations

Plasma levels of corticosterone were determined using a commercially available enzyme-linked immunosorbent assay (IBL, Minnesota). TNF-*α* levels were determined using a commercially available enzyme-linked immunosorbent assay (R&D Systems, Minneapolis, MN).

### Real-time PCR analysis

One microgram of total RNA was reverse-transcribed to cDNA with Superscript II reverse transcriptase and poly dT priming (Life Technologies, Rockville, MD). Quantitative RT-PCR was carried out on a LightCycler (LightCycler 480, Roche Diagnostics, Mannheim, Germany) and a standard LightCycler amplification cycle protocol was established for each gene. The primers are: β-actin: 5′- CGCCCTAGGCACCAGGGTG-3′ (sense), 5′- GCTGGGGTGTTGAAGGTCTCAAA-3′ (antisense) [26]; iNOS: 5′- AGACGCACAGGCAGAGGT-3′ (sense), 5′-AGGCACACGCAATGATGG-3 (antisense); eNOS: 5′-CTGCTGCCCGAGATATCTTC-3′ (sense), 5′- CAGGTACTGCAGTCCCTCCT-3′ (antisense) [27]; The first segment of the amplification cycle consisted of a denaturation program of 95°C for 10 min. The second segment consisted of denaturation (95°C for 15 s), primer annealing (58°C for 5 s), elongation (72°C for 10 s) and quantification program repeated for 40 cycles. The third segment consisted of a melting curve program (95°C for 0 s, 57°C for 15 s and a linear temperature transition at 0.05°C/s from 57°C to 95°C with continuous fluorescence acquisition). The final segment consisted of a cooling program to 4°C. An internal housekeeping gene, β-actin, was used to normalize differences in RNA isolation, RNA degradation, and the efficiencies of the RT. Abundance of mRNA was determined by real time RT-PCR normalized to abundance of β-actin mRNA. LightCycler analysis software (Roche Diagnostics, Mannheim, Germany) allowed the quantitative analysis.

### Experimental design

**Figure 1:**
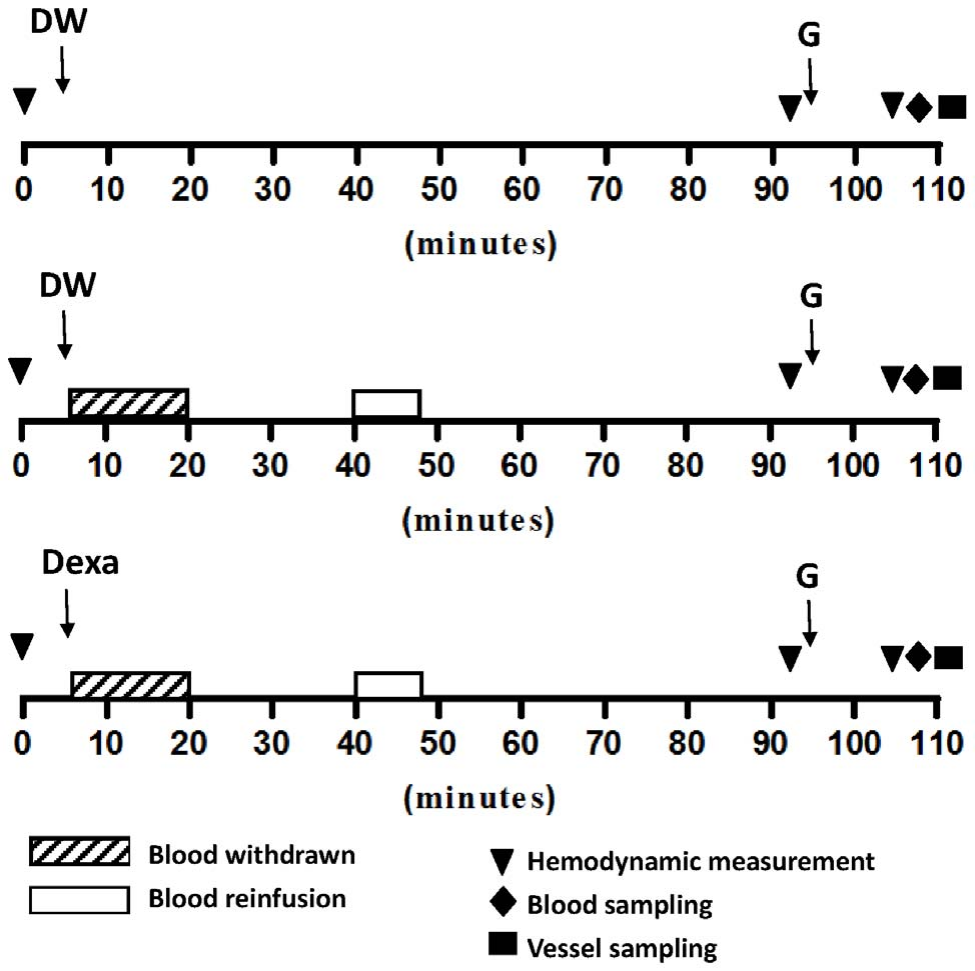
Experimental design. G: glypressin; DW: distilled water (vehicle); Dexa: dexamethasone (3 or 5 mg/kg).

The experimental design is shown in Figure 1. Rats were anesthetized with ketamine hydrochloride (100 mg/Kg, i.m.) (time 0). After baseline MAP, HR and PP measurements (+5 minute), blood was withdrawn in 15 minutes with the amount of (1) 35% of total blood volume in PVL rats and (2) 25% of total blood volume in adrenalectomized PVL rats at constant rates (+20 minute). Vehicle (distilled water, DW) or dexamethasone (3 or 5 mg/kg) was injected intravenously upon the onset of hemorrhage. After a 20-min stabilization (+40 minute), 50% of the withdrawn blood was re-infused for 7.5 min at the same rate (+47.5 minute). The procedure was modified based on the previously published work.^24^ Blood loss was estimated using the total blood volume as 54 ml/Kg. A blood infusion/withdrawal pump (model SP 210 iw, World Precision Instruments, Sarasota, FL) was used via a PE-50 catheter connected to the right carotid artery. In the without-hemorrhage group, no blood was manipulated during the course. Forty-five minutes later (+92.5 minute), the second hemodynamic measurement was performed, followed by glypressin (0.07 mg/kg) infusion with the pump via the right jugular vein. Ten minutes after glypressin administration (+105 minute), the third hemodynamic study was performed. Then blood was withdrawn from the inferior vena cava for TNF-α measurement and AA and SMA were dissected for real-time PCR analysis of the NOS expressions.

### Study protocol

#### (1) PVL rats

In the first series, PVL rats with- or without-hemorrhage received ACTH stimulation test. In the second series, rats were divided into without-(PHN) or with-hemorrhage groups injected with vehicle (PHV) or dexamethasone (3 mg/kg, PHD). The dose of dexamethasone was decided according to the literature, capable of inhibiting NOS-mediated vascular hyporeactivity in hemorrhagic shock.^6^ The following parameters were evaluated: (i) hemodynamic responses to glypressin, (ii) serum levels of TNF-α, and (iii) AA and SMA NOS mRNA expressions.

#### (2) PVL and adrenalectomized rats

To exclude the potential interference exerted by endogenous adrenal hormone in interpreting the effects of exogenous glucocorticoid supplement, PVL rats received bilateral adrenalectomy and then were divided into without- (PAHN) or with-hemorrhage groups with vehicle (PAHV)-, dexamethasone (3 mg/kg, PAHD3)-, or high-dose dexamethasone (5 mg/kg, PAHD5) injections. The same parameters as above were evaluated.

### Reagents

Porcine corticotropin (ACTH (1–39)) and dexamethasone were purchased from Sigma-Aldrich corporation (St Louis, MO). Glypressin was purchased from Ferring Company (Kiel, Germany).

### Statistical Analysis

Results are expressed as means ± SE. Statistical analyses were performed using one way ANOVA or unpaired Student's *t* test when appropriate. A two-tailed P value of <0.05 was considered statistically significant.

## Results

### Baseline body weight and hemodynamics in different experimental groups

Table 1 shows the baseline body weight (BW) and hemodynamic parameters. They were not significantly different among subgroups of PVL rats and of adrenalectomized PVL rats. PVL rats had significantly higher levels as compared with those of the adrenalectomized PVL rats (BW: 330±7 vs. 292±3 g, P<0.001; MAP: 115.7±2.7 vs. 85.9±3.4 mmHg, P<0.001; PP: 13.4±0.4 vs. 10.5±0.7 mmHg, P = 0.001; HR: 342±11 vs. 313±7/min, P = 0.023).

**Table 1:** Body weight and baseline hemodynamics in different groups.

	n	BW (g)	MAP (mmHg)	PP (mmHg)	HR (/min)
ACTH-H (−)	21	338±4	114.9±2.8	14.9±0.5	344±8
ACTH-H (+)	19	338±7	117.1±2.6	15.3±0.5	336±8
PHN	8	331±6	120.6±5.2	14.0±0.4	352±20
PHV	6	315±13	115.8±5.4	13.0±0.9	331±21
PHD	7	341±18	109.9±2.4	13.0±0.7	340±17
PAHN	7	297±7	83.9±4.9	10.5±1.7	310±7
PAHV	9	287±5	84.5±9.0	10.3±1.5	303±17
PAHD3	7	296±6	93.5±4.9	10.9±1.5	339±14
PAHD5	11	289±6	86.1±5.8	10.4±1.1	303±12

MAP: mean arterial pressure; PP: portal pressure; HR: heart rate;

PAHN: without hemorrhage; PAHV, PAHD3, PAHD5: hemorrhage groups treated with vehicle, dexamethasone 3 mg/kg, or 5 mg/kg, respectively.

P>0.05 between ACTH H (−) and H (+) groups, among PHN, PHV, PHD groups, and among PAHN, PAHV, PAHD3, PAHD5 groups.

### Corticosterone synthesis in response to ACTH stimulation

**Figure 2:**
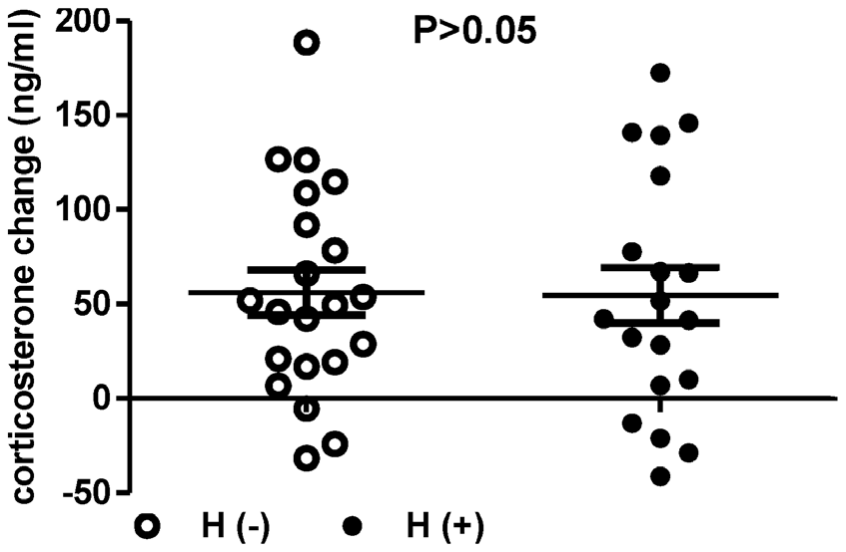
ACTH-induced corticosterone changes in PVL rats without- (H(-), n = 21) or with-hemorrhage (H(+), n = 19). There was no significant difference (P>0.05).

Figure 2 shows that although the with-hemorrhage PVL rats tended to have higher corticosterone levels before and after ACTH injection as compared with the without-hemorrhage PVL rats, they did not reach the statistical significance (with- vs. without-hemorrhage (ng/ml): before ACTH: 336.7±21.0 vs. 288.3±25.1, P = 0.153; after ACTH: 391.3±14.7 vs. 344.4±26.8, P = 0.145). The changes of corticosterone levels elicited by ACTH were not significantly different (54.6±14.7 vs. 56.1±12.1, P = 0.939), either.

### (1) PVL rats

#### Hemodynamic responses to glypressin

##### MAP

**Figure 3:**
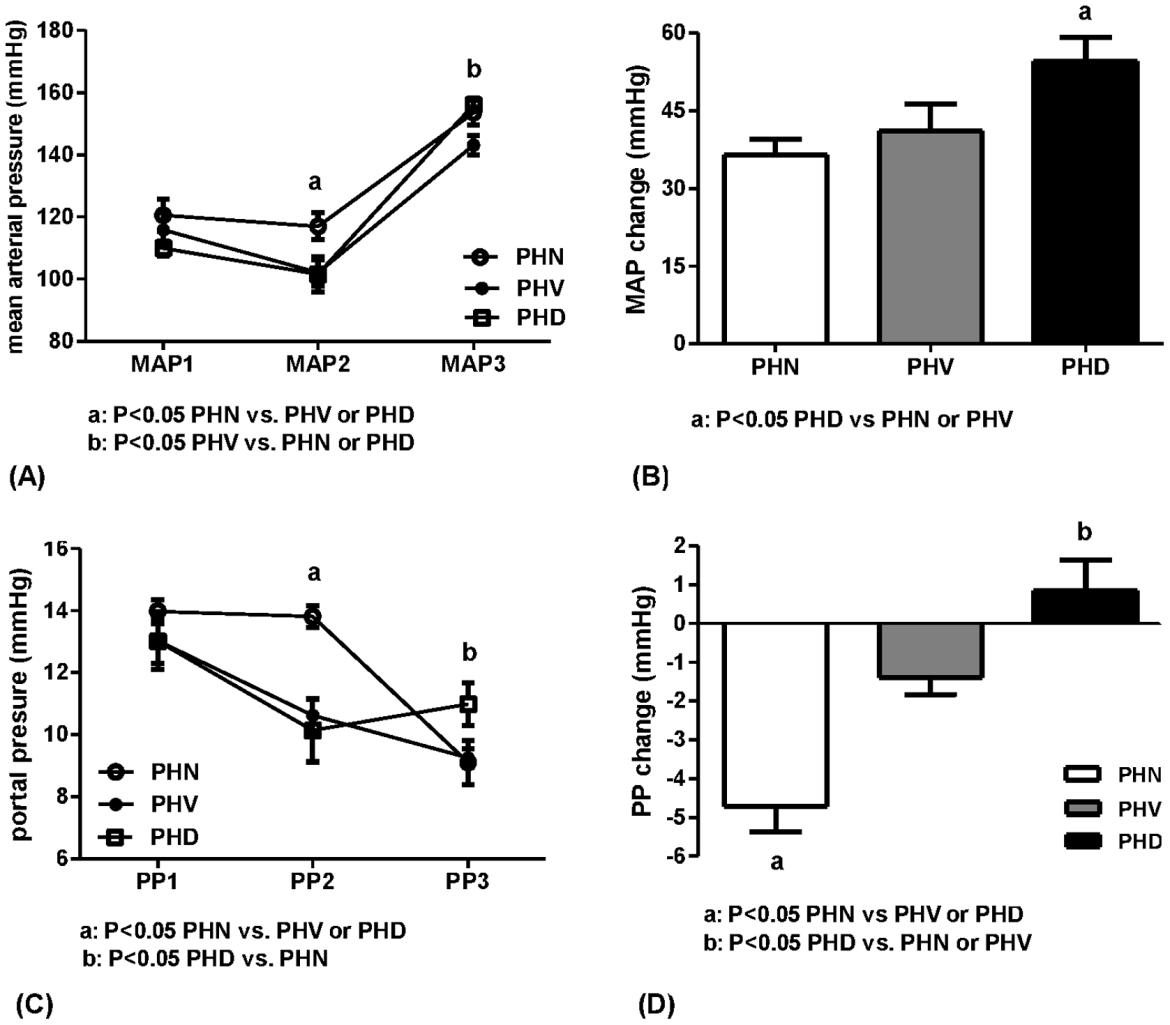
Glypressin-induced hemodynamic changes in PVL rats. (A) Chronological changes of MAP in PVL rats without-(PHN, n = 8) or with-hemorrhage treated with vehicle (PHV, n = 6) or dexamethasone 3 mg/kg (PHD, n = 7). MAP1: baseline; MAP2: 45 minutes after hemorrhage-transfusion; MAP3: 10 minutes after glypressin. PHN group had significantly higher MAP2 (P<0.05 vs. PHV or PHD) and PHV group had lower MAP3 (P<0.05 vs. PHN or PHD). (B) PHD group had higher glypressin-induced MAP change (MAP3-MAP2) (P<0.05 vs. PHN or PHV). (C) Chronological changes of PP. PHN group had higher PP2 (P<0.05 vs. PHV or PHD) and lower PP3 (P<0.05 vs. PHD). (D) PHN group had a more prominent PP reduction (PP3-PP2, P<0.05 vs. PHV or PHD).

Figure 3(A) depicts that forty-five minutes after the hemorrhage period, the MAP2 (mmHg) of PVL rats was: 117.0±4.3 (PHN), 102.1±4.2 (PHV), and 101.6±5.6 (PHD), respectively, significantly higher in the PHN group than in the other two groups (P = 0.042 vs. PHV and 0.030 vs. PHD, respectively). Ten minutes after glypressin injection, the MAP3 was 153.5±3.9, 143.1±3.0, and 156.0±2.1, respectively, significantly lower in the PHV group (P = 0.038 vs. PHN and 0.015 vs. PHD, respectively). Figure 3(B) demonstrated that the glypressin-elicited MAP increase was 36.5±3.0, 41.1±5.3, 54.5±4.7, respectively, significantly higher in the PHD group (P = 0.006 vs. PHN and 0.046 vs. PHV, respectively).

##### PP

Figure 3(C) reveals that the PP2 (mmHg) of PVL rats was: 13.8±0.3 (PHN), 10.6±0.5 (PHV), 10.1±1.0 (PHD), respectively, significantly higher in the PHN group (P = 0.004 vs. PHV and 0.001 vs. PHD, respectively). PP3 was 9.1±0.7, 9.3±0.3, 11.0±0.7, respectively, in which the PHN group had a significantly lower PP than the PHD group (P = 0.045). Figure 3(D) discloses that the glypressin-elicited PP change was −4.7±0.6, −1.4±0.5, and 0.8±0.8, respectively. The PHN group had a more significant reduction of PP than the other two bleeding groups (P = 0.003 vs. PHV and <0.001 vs. PHD, respectively).

#### Serum TNF-α concentration

**Figure 4:**
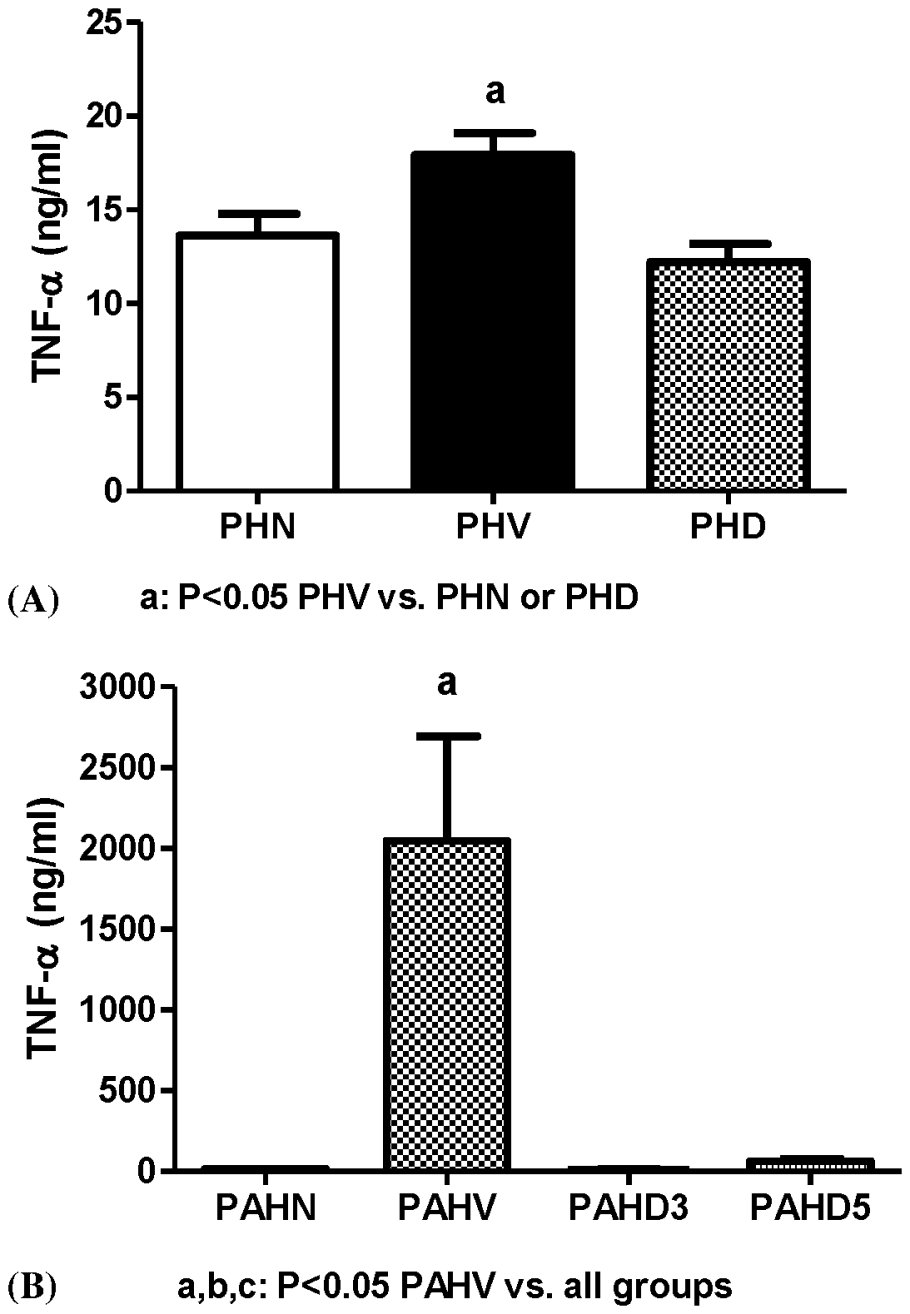
TNF-α concentrations in (A) PVL (PHN, PHV, PHD) and (B) adrenalectomized PVL (PAHV, PAHV, PAHD3, PAHD5) groups. (A): TNF-α level was significantly higher in PHV group (P<0.05 vs. PHN) and was reduced by dexamethasone (P<0.05 PHV vs. PHD). (B): PAHV group had significantly higher TNF-α level (P<0.05 vs. all groups).

Figure 4(A) reveals that the TNF-α (ng/ml) concentration was significantly higher in the PHV group as compared with the PHN group (17.9±1.2 vs.13.6±1.1, P = 0.016), which could be significantly reduced by dexamethasone (12.2±1.0, P = 0.006 vs. PHV).

#### Vascular NOS expressions

##### Abdominal aorta

**Figure 5:**
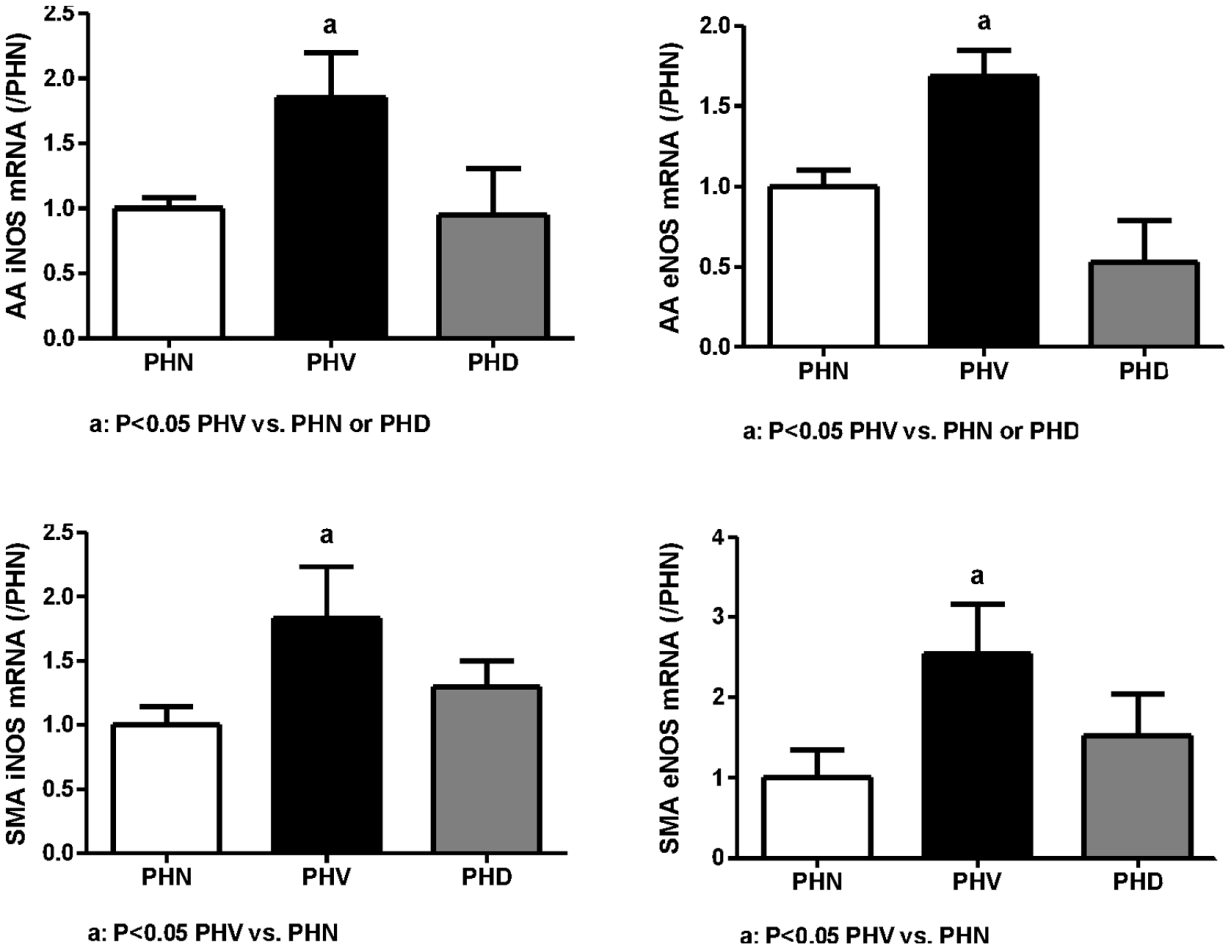
The vascular NOS mRNA expressions in PVL rats. AA: abdominal aorta; SMA: superior mesenteric artery; iNOS: inducible nitric oxide synthase; eNOS: endothelial NOS. PHV group had higher AA and SMA iNOS, eNOS expressions (P<0.05 vs. PHN), which were down-regulated by dexamethasone in AA (P<0.05 vs. PHD), but not in SMA.

Figure 5 reveals that the iNOS expressions in PHV and PHD groups were (relative ratio to PHN): 1.8551±0.3387, and 0.9499±0.3552, respectively. The PHV group had a higher iNOS expression than that of the PHN group (P = 0.022), which was significantly suppressed by dexamethasone (P = 0.025 vs. PHV). The eNOS expressions were: 1.6853±0.1643 and 0.5256±0.2605, respectively. The PHV group had a higher eNOS expression than that of the PHN group (P = 0.009), which was also significantly suppressed by dexamethasone (P<0.001 vs. PHV).

##### Superior mesenteric artery

The iNOS expressions of PHV and PHD groups were (relative ratio to PHN): 1.8278±0.4073, and 1.2945±0.2053, respectively. The PHV group had a significantly higher iNOS expression than that of the PHN group (P = 0.025), which was not significantly influenced by dexamethasone (P>0.05). The eNOS expressions were: 2.5447±0.6151 and 1.5256±0.5205, respectively. The PHV group had significantly higher eNOS expression than that of the PHN group (P = 0.030), which was not significantly suppressed by dexamethasone (P>0.05), either.

### (2) PVL+adrenalectomy rats

#### Hemodynamic responses to glypressin

##### MAP

**Figure 6:**
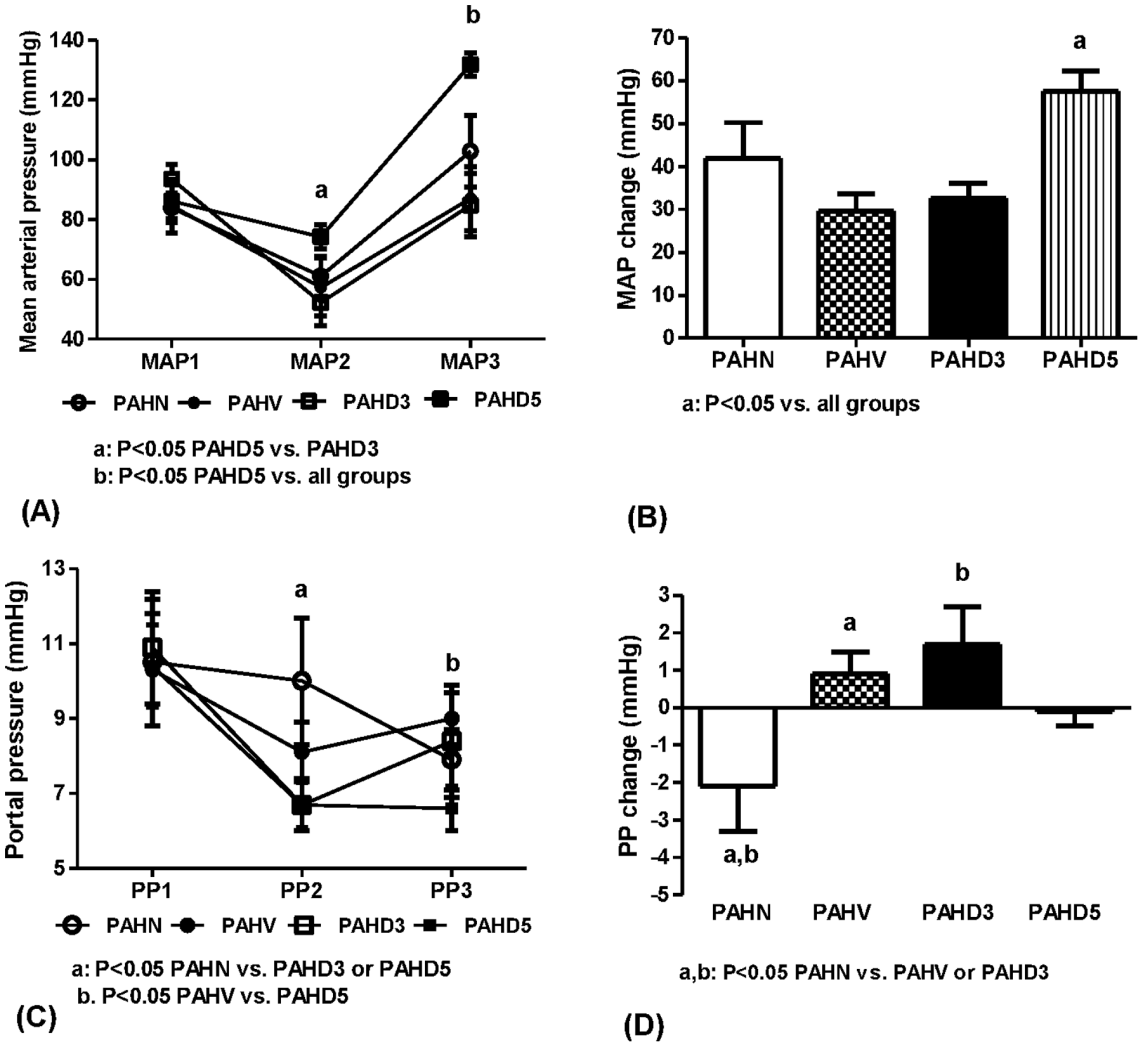
Glypressin-induced hemodynamic changes in adrenalectomized PVL rats. (A) Chronological MAP changes in adrenalectomized PVL rats without-(PAHN, n = 7) or with-hemorrhage treated with vehicle (PAHV, n = 9), dexamethasone 3 (PAHD3, n = 7), or 5 mg/kg (PAHD5, n = 11). PAHD5 group had higher MAP2 than PAHD3 group and higher MAP3 than all groups (P<0.05); (B) PAHD5 group had higher MAP change (P<0.05 vs. all groups). (C) Chronological changes of PP. PAHN group had higher PP2 (P<0.05 vs. PAHD3 or PAHD5) and PAHD5 group had lower PP3 (P<0.05 vs. PAHV); (D) PAHN group had more prominent PP reduction (P<0.05 vs. PAHV or PAHD3).

Figure 6(A) reveals that the MAP2 (mmHg) was: 61.0±6.6 (PAHN), 57.3±9.7 (PAHV), 52.3±7.8 (PAHD3), and 74.3±4.0 (PAHD5), respectively. MAP2 of the PAHD5 group was significantly higher than that of the PAHD3 group (P = 0.036). MAP3 was 102.8±11.9, 87.0±10.7, 84.8±10.5, and 131.8±4.0, respectively, which was significantly higher in the PAHD5 group than in the other three groups (P = 0.030 vs. PAHN, 0.001 vs. PAHV, and 0.001 vs. PAHD3, respectively). Figure 6(B) shows that the glypressin-induced MAP elevation was 41.8±8.4, 29.6±4.1, 32.5±3.6, and 57.5±4.7, respectively, which was also significantly higher in the PHD5 group than the other three groups (P = 0.043 vs. PAHN, <0.001 vs. PAHV, and 0.002 vs. PAHD3, respectively).

##### PP

Figure 6(C) depicts that the PP2 (mmHg) was: 10.0±1.7 (PAHN), 8.1±0.8 (PAHV), 6.7±0.7 (PAHD3), and 6.7±0.6 (PAHD5), respectively. PP2 of the PAHN group was significantly higher than those of the PAHD3 and PAHD5 groups (P = 0.038 vs. PAHD3 and 0.023 vs. PAHD5, respectively). PP3 was 7.9±0.8, 9.0±0.7, 8.4±1.5, and 6.6±0.6, respectively, in which the PHD5 group had a significantly lower PP than that of the PAHV group (P = 0.042). Figure 6(D) discloses that glypressin-induced PP change was −2.1±1.2, 0.9±0.6, 1.7±1.0, and −0.1±0.4, respectively. The PAHN group had a significantly more PP reduction than those of the PAHV and PAHD3 groups (P = 0.012 vs. PAHV and 0.003 vs. PAHD3, respectively) but not different from the PAHD5 group (P = 0.077).

#### Serum TNF-α concentration

Figure 4(B) shows that the TNF-α(ng/ml) concentrations were 46.6±10.8 (PAHN), 2045.2±649.2 (PAHV), 9.4±0.7 (PAHD3), and 61.0±14.5 (PAHD5), respectively. The PAHV group had a significantly higher TNF-α level than the other three groups (P = 0.006 vs. PAHN, <0.001 vs. PAHD3, and 0.001 vs. PAHD5, respectively).

#### Vascular NOS expressions

##### Abdominal aorta

**Figure 7:**
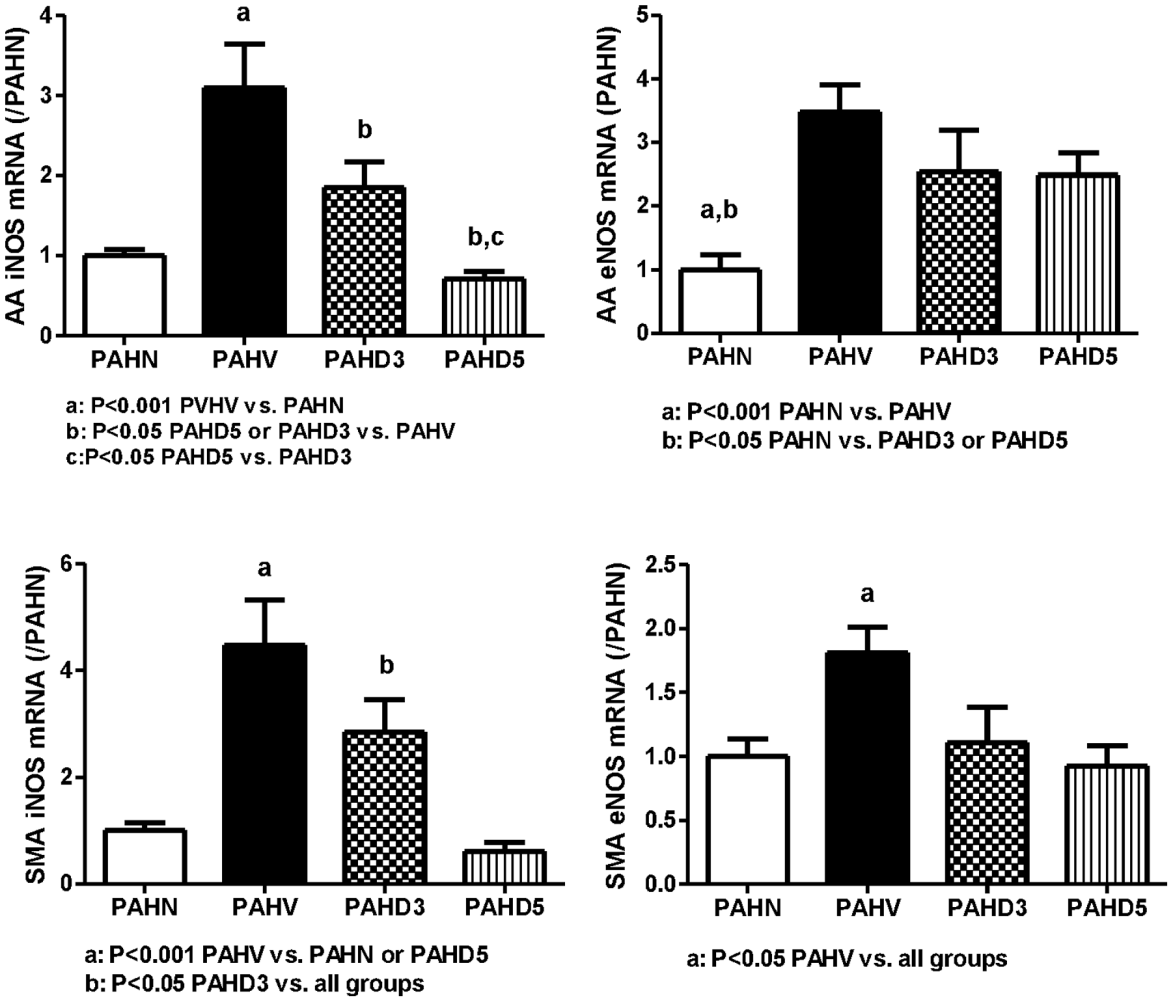
The vascular NOS mRNA expressions in adrenalectomized PVL rats. PAHV group had higher AA iNOS, eNOS expressions (P<0.001 vs. PAHN), in which iNOS was down-regulated by dexamethasone, especially 5 mg/kg (P<0.05, PAHD5 vs. PAHD3). PAHV group had higher SMA iNOS expression (P<0.001 vs. PAHN), which was down-regulated by dexamethasone, especially 5 mg/kg. PAHV group had higher SMA eNOS expression (P<0.05 vs. PAHN), which was down-regulated by dexamethasone (P<0.05 vs. PAHD3 or PAHD5).

Figure 7 reveals that the iNOS mRNA expressions in PAHV, PAHD3 and PAHD5 groups were (relative ratio to PAHN): 3.1006±0.5473, 1.8516±0.3140, and 0.7093±0.0974, respectively. The PAHV group had a higher iNOS expression than that of the PAHN group (P<0.001), which was significantly down-regulated by either dose of dexamethasone (PAHD3 vs. PAHV: P = 0.015; PAHD5 vs. PAHV: P<0.001, respectively). Moreover, high dose dexamethasone (5 mg/kg) exerted a more prominent iNOS inhibition than that of the 3 mg/kg dexamethasone (P = 0.03). The eNOS expressions were: 3.4792±0.4213, 2.5412±0.6579, and 2.4834±0.3525, respectively. The PAHV group had a significantly higher eNOS expression than that of the PAHN (P<0.001) group and tended to be down-regulated in PAHD3 and PAHD5 groups but not statistically different (P>0.05).

##### Superior mesenteric artery

The iNOS expressions in PAHV, PAHD3 and PAHD5 groups were (relative ratio to PAHN): 4.4840±0.8498, 2.8489±0.6033, and 0.6021±0.1787, respectively. The PAHV group had a significantly higher iNOS expression than that of the PAHN group (P<0.001), which was significantly down-regulated by either dose of dexamethasone (PAHD3 vs. PAHV: P = 0.045; PAHD5 vs. PAHV: P<0.001, respectively). High dose dexamethasone exerted a significantly stronger iNOS inhibition than that of the lower dose (P = 0.007). The eNOS expressions were: 1.8102±0.1989, 1.1073±0.2742, and 0.9284±0.1564, respectively. The PAHV group had a significantly higher eNOS expression than that of the PAHN group (P = 0.007), which was significantly suppressed by either dose of dexamethasone (PAHD3 vs. PAHV: P = 0.018; PAHD5 vs. PAHV: P = 0.003, respectively).

## Discussion

In the current study, PVL rats with adrenalectomy showed lower BW, MAP, PP, and HR as compared with those without adrenalectomy. Glucocorticoid is essential for body weight and hemodynamic homeostasis and its deficiency is accompanied by low blood pressure [28]. A previous survey had also found a poor body weight gain and the tendency of decreased resting arterial blood pressure in the adrenalectomized rats, which could be restored by corticosterone [29]. In another study, adrenalectomy induced decreased systolic and diastolic blood pressure, stroke volume, and body weight in Pekin ducks. A synthetic glucocorticoid, betamethasone, prevented the hypotension and body weight loss [30].

It has been well documented that patients with the normal HPA axis develop sufficiently elevated circulating cortisol levels in response to severe illness [31]. Our data demonstrated that bleeding PVL rats, as compared with those without hemorrhage, did not exert an enhanced corticosterone response to ACTH stimulation. This provides the evidence for the first time that in portal hypertension, hemorrhage without significant evidence of sepsis is still featured by CIRCI. One possibility is that the increased circulating corticosterone levels following hemorrhage and resuscitation might reduce adrenal responsiveness to corticotropin stimulation [25]. Actually, the bleeding PVL rats tended to have higher corticosterone levels, either before or after ACTH administration. On the other hand, compatible with our data, hemorrhage elicits cytokines release such as TNF-α [32]. Although it has not been tested in hemorrhagic shock previously, high levels of inflammatory cytokines directly inhibit cortisol synthesis and depress the magnitude of cortisol response to corticotropin [33]. In fact, cirrhotic patients and animals are characterized by increased levels of endotoxin and inflammatory cytokines, which may contribute to NO overproduction, hemodynamic impairment [34], [35], and potentially to adrenal dysfunction. The increased plasma levels of TNF-α have also been found by our group in portal hypertensive rats [36].

In this study, dexamethasone ameliorated the elevated circulating TNF-α concentrations after hemorrhage, both in PVL rats with or without adrenalectomy. This is compatible with the previous study, showing that corticosteroids inhibit TNF-α production, both in murine and rat macrophages [37]. Indeed, hemorrhage has been reported to induce an increase in serum TNF-α [38], followed by NOS activation and vascular hypo-reactivity [6]. A previously survey indicated that dexamethasone treatment ameliorated the hemodynamic adverse effects elicited by TNF in adrenalectomized CD-1 mice [39].

It has been reported that glucocorticoid potentiated pressor response to arginine vasopressin in rat [40]. This potentiation was prevented by an antiglucocorticoid agent, suggesting the major role of glucocorticoid in this action. To get rid of the confounding influences exerted by endogenous adrenal hormone, PVL rats received adrenalectomy. The data indicated that vehicle-treated bleeding adrenalectomized PVL rats had significantly poorer systemic and splanchnic vascular responsiveness to glypressin as compared with high- dose dexamethasone-treated ones. This is supported by the notion that the lack of glucocorticoid led to a reduced vascular reactivity [28]. Others have established that adrenalectomy is associated with reduced vasocontractile responses to norepinephrine and α-adrenergic receptor agonists [41]. Such a decreased vascular sensitivity in the adrenalectomized rats could be restored by corticosterone [29], but not the mineralocorticoid aldosterone [42]. However, the hemodynamic responses to glypressin in PVL rats without or with adrenalectomy could not be compared directly in this study, since they were subjected to different magnitudes of blood loss (35% or 25% of total blood volume loss, respectively): In preliminary experiments, all adrenalectomized PVL rats with 35% blood loss died during the hemorrhage-transfused stage that a less drastic amount of blood loss (25%) was applied. Nevertheless, this is not deviated from the main theme of this study. Furthermore, the finding demonstrated that adrenalectomized PVL rats could only tolerate less blood loss since they failed to maintain hemodynamic homeostasis at the same magnitude of blood loss as PVL rats.

It is worth noting that AA iNOS and eNOS were up-regulated in bleeding PVL rats, which could be down-regulated by dexamethasone. Furthermore, adrenalectomized PVL rats responded to high dose dexamethasone with more prominent and significant MAP elevation and iNOS down-regulation. It has been demonstrated that the vasodilatation, sustained hypotension, and hyporeactivity to vasoconstrictors characterizing the circulatory failure in endotoxin shock are mediated by enhanced NO release [43]. It has been reported that dexamethasone elevated blood pressure via the suppression of vascular eNOS expression [44]. The capability of dexamethasone to suppress iNOS expression [45] may also contribute to the reversal of systemic shock.

Consistent with the previously described splanchnic hyposensitivity in PVL rats with acute hemorrhage [46], our data showed that bleeding PVL rats had a less glypressin-induced PP reduction as compared with those without hemorrhage. The glypressin-induced PP reduction depends mainly on the splanchnic vasoconstriction. We have also previously reported that the SMA NOS overexpression is related to the splanchnic hyposensitivity [47]. Consistent with this notion, this study identified that in adrenalectomized PVL rats, the enhanced SMA NOS expressions and less PP reduction to glypressin after hemorrhage were reversed by high-dose dexamethasone. However, dexamethasone 3mg/kg use in PVL rats did not correct the splanchnic hyposensitivity and SMA NOS over-expressions. Taken together, the results suggest a dose-dependent effect of dexamethasone in portal hypertensive status with hemorrhage. The current results implicate that in clinical setting, patients with portal hypertension with acute hemorrhage prone to suffer from profound systemic shock and splanchnic hyposensitivity related to adrenal insufficiency, which can be reversed by adequate corticosteroid supplement. Nevertheless, further dose-finding clinical studies are required.

In conclusion, bleeding portal hypertensive rats exerted relative corticosteroid insufficiency featured by insignificant corticosterone response to corticotropin compared with those without hemorrhage. The MAP elevation, circulating TNF-α level reduction and AA NOS expressions downregulation elicited by dexamethasone and the higher dose required for adrenalectomized PVL rats suggested the importance of adequate glucocorticoid supplement to correct hemorrhagic shock. Splanchnic hyposensitivity to glypressin and SMA NOS up-regulations during hemorrhage could be reversed in adrenalectomized PVL rats by high dose dexamethasone, indicating the potential role of glucocorticoid to control portal pressure and gastroesophageal variceal hemorrhage in clinical practice.
